# Effect of Root Repair Materials and Bioactive Glasses on Microhardness of Dentin

**DOI:** 10.22037/iej.v13i3.20565

**Published:** 2018

**Authors:** Olinto Santos Cardoso, Meire Coelho Ferreira, Edilausson Moreno Carvalho, Paulo Vitor Campos Ferreira, José Bauer, Ceci Nunes Carvalho

**Affiliations:** a *School of Dentistry, CEUMA University, São Luis, Maranhão, Brazil; *; b * Discipline of Dental Materials, School of Dentistry, Federal University of Maranhão, Maranhão, Brazil; *; c * Department of Dental Materials, School of Dentistry, University of Campinas, São Paulo, Brazil*

**Keywords:** Bioactive Glass 45S5, Dental Materials, Hardness Test, Microhardness Tests, Root Canal Filling Materials

## Abstract

**Introduction:**

The use of bioactive glasses to re-establish or increase mechanical properties of the root dentin may be an interesting alternative. The aim of this study was to evaluate the effect of root repair materials and bioactive glasses on the microhardness of human root dentin.

**Methods and Materials:**

Sixty-four sectioned palatal roots of human molars were prepared and two slices were obtained of the middle third of each root (one corresponding to the control group, without treatment, and the other to the experimental group). The pairs of slices were randomly divided into four groups (*n*=16). The root canal of experimental slices were filled with one of the following materials: mineral trioxide aggregate (Angelus MTA, Angelus, Londrina, Paraná, Brazil), EndoSequence Root Repair Material (ERRM, Brassler, Savannah, GA, USA), Bioglass (45S5) and an experimental niobophosphate glass (NbG). The specimens were stored in an oven at 37^º^C, in an environment with 100% humidity for 60 days. The specimens were subjected to a microhardness test. Four indentations were made at a distance of 20 µm from the root canal lumen. For microhardness analysis, comparing the experimental groups and their respective controls, the Student’s*-t* test was applied. For comparison of the percentage increase in microhardness between the groups, the data were statistically analyzed by using One-way ANOVA and Tukey’s test. The level of significance was set at 0.05.

**Results:**

All the materials significantly increased the dentin microhardness values (*P*<0.05). MTA showed a higher increase in microhardness (94.8±42.7%), similar to that of EndoSequence (62.3±39.9%). The 45S5 (46.5±30.0%) and NbG (53.8±31.3%) showed the lowest percentages of increase in microhardness, but were statistically similar to those of EndoSequence.

**Conclusion:**

All the materials tested were capable of increasing root dentin microhardness.

## Introduction

The first calcium silicate-based hydraulic cement patented for endodontic use was mineral trioxide aggregate (MTA) [1], which is considered as the gold standard for many clinical applications due to its excellent sealing capacity, biocompatibility, regenerative capacity and antibacterial properties [2]. MTA has been widely used as a reparative material in cases of perforation, retrofilling and apexification or for direct pulp capping [3]. However, it still has some disadvantages such as a long setting time, being difficult to handle and the possibility of staining the dental structures [4].

Silicate-based biomaterials are a new generation of biological cements that consist of hydraulic calcium phosphate and silicates, with the expectation that hydration processes would improve the mechanical properties and biocompatibility of the cement [5]. An example of these new generation cements is EndoSequence Root Repair Material Paste (ERRM, Brassler, Savannah, GA, USA). Many reports have indicated that this type of material is capable of producing an appetite-rich superficial layer after contact with simulated body fluids [6-9]. Another way, ERRM may be more toxic to fibroblasts than MTA [10].

Bioactive glasses have been proposed for application with the purpose of promoting dentin remineralization by the precipitation of calcium phosphate in the medium [11]. These characteristics make these biomaterials interesting for use in teeth that suffer some traumatism, especially in cases requiring the treatment of immature or weakened teeth [12]. Bioactive glasses 45S5 are better known in applications as biomaterials, because their structure is closer to that of the mineral portion of bony and dental tissues [13]. However, the disadvantages of these glasses is their low chemical durability, another way, this durability may be improved with the addition of niobium oxide [14], which is not only biocompatible, but is capable of increasing the radiopacity and microhardness when these glasses are incorporated into endodontic cements [15] and adhesive system [16].

In traumatized or weakened teeth, it would be interesting to use root repair materials that could re-establish or increase the root dentin microhardness [17]. On the other hand, there are no studies in the literature that evaluate the influence of these bioactive glasses, used as repair of the root canal, on the microhardness of the dentin.

Therefore, the aim of this study was to evaluate the effect of different root repair materials on root dentin microhardness. The null hypothesis tested was that none of the materials tested would alter the microhardness of radicular dentin.

## Materials and Methods

***Selection and preparation of teeth***

For sample size calculation for comparing the mean microhardness values (KHN) among the groups, the following parameters were considered: a level of confidence of 95%; power of 80%, standard deviation of 5 [18] and a minimum difference to be detected among groups of 5 points in the mean microhardness. The sample size calculated per group was 16. 

After approval from the Ceuma University Research Ethics Committee (protocol number 1.750.974/2016) a selection was made of 64 human maxillary first molar teeth, with completely formed apices, extracted for various reasons. The organic matter was removed from the root surface by means of curettes. The teeth were stored in 0.1% thymol at 4^°^ C and used within 6 months after extraction. The specimens showed radiographically absence of any sign of diffuse or localized calcification, internal resorption or previous endodontic treatment. 

The palatal roots (used for the study) were sectioned perpendicular to the long axis of the tooth, immediately below the enamel-cementum junction, by means of a cutting machine (Isomet 1000 Precision Saw Buehler Ltd, Lake Bluff, IL, USA) under constant water irrigation. The working length (WL) was determined for each tooth, by introducing a #10 K-file (Maillefer, Ballaigues, Switzerland) into the root canal until the tip of the file was visualized in the apical foramen with the aid of a stereoscopic lens under ×25 magnification (Baush, Lomb, Rochester, USA), and then subtracting 1 mm from the measurement obtained.

The canals were emptied with K-files up to #15, with 1.0% sodium hypochlorite solution (Fórmula e Ação, São Paulo, SP, Brazil). For canal instrumentation, 10 mL of 1% sodium hypochlorite (pH=11) and 3 mL of EDTA 17% (Fórmula e Ação, São Paulo, SP, Brazil) were used, and performed with Reciproc R50 (50/0.05) (VDW, Munich, Germany).

***Sample preparation***

**Figure 1:**
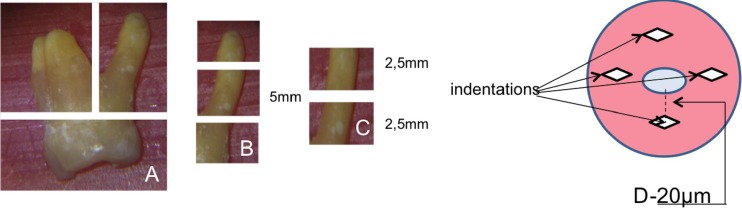
Schematic illustration of the root sections and demarcations of indentations made in the specimens

After biomechanical preparation, a 5 mm section on the middle third of each root was sectioned, and this was sectioned into two segments with a thickness of 2.5 mm, with the use of an Isomet cutting machine (Isomet 1000 Precision Saw Buehler Ltd.). One section was used for inserting the reparative material, and the other was considered the control of the sample (Figure 1).

The specimens were randomly divided into four groups, with variation of the type of root repair material used (*n*=16) as follows: ERR (EndoSequence Root Repair Material Paste, Brasseler USA, Savannah, GA, USA); Ângelus MTA (Ângelus Odontológica, Londrina, Paraná, Brazil); 45S5 (Sylc, OSspray Ltd, London, UK) in distilled water used as the vehicle; NbG–Experimental niobophosphate glass in distilled water used as the vehicle. The NbG was obtained by the fusion of chemical precursors in an electric furnace, according to previous studies [14, 19].

To standardize the consistency of the paste for the different bioactive glasses used, for each preparation was used the same quantity of powder and liquid in the proportion of 2:1; 4 g glass to 2 mL distilled water. 

The commercial root repair materials were manipulated in accordance with the manufacturers’ instructions. The root canals of the sections were filled with the syringe and tip made available by the manufacturer itself, in the case of ERR and with the aid of an insertion spatula for the other groups. After filling with the materials, all specimens were stored in an oven at 37^º^C, in an environment with 100% humidity for 60 days.

After the storage period, the specimens were embedded in PVC tubes and acrylic resin (TDV, Pomerode, SC, Brazil) and the dentin surface was abraded with abrasive papers of decreasing granulations: 400#, 600#, 1200#; and 2 and 3 µm diamond paste (Diamond Excel, FGM, Joinville, SC, Brazil) with polishing cloths. 

***Microhardness analysis***

Dentin microhardness was measured with a Knoop indenter under ×40 magnification (Shimadzu HMV-2000, Kyoto, Japan) with a load of 10 g and 15 sec dwell time. On each sample, four indentations were made at a distance of 20 µm from the root canal lumen (Figure 1). 

The microhardness value of each specimen was obtained by calculating the mean value of the results of four indentations. 

***Statistical analysis***

Statistical analysis was performed using the Statistical Package for Social Sciences (SPSS for Windows 21.0, SPSS Inc. Chicago, IL, USA). The Shapiro-Wilk and Levene tests were performed first, showing normality of the data distribution and the equality of the variances. Subsequently, statistical analysis was performed using the appropriate statistical tests. For microhardness analysis, comparing the experimental groups and their respective controls, the paired Student’s*-t* test was applied.

For comparison of the percentage increase in microhardness among the groups, the one-way ANOVA and Tukey’s test for difference between the means of each contrast were used. The level of significance was set at 0.05. 

## Results

The mean KHN values and standard deviations of root dentin before and after treatments with the materials MTA, ERR, 45S5, NbG are described in Table 1. 

The one-way analyses statistical showed that dentin KHN values increased after treatment with all the materials tested when compared with their controls (*P*<0.05). 

The percentage (%) of increase in dentin microhardness is described in Table 2. MTA showed the highest percentage increase in microhardness, but showed no statistically significant difference in comparison with ERR. Both bioactive glasses, 45S5 and NbG showed the lowest percentage of increase in microhardness, but showed no statistical difference in comparison with ERR (*P*>0.05). 

## Discussion

The present study showed that commercial root repair materials and bioactive glass pastes are capable of increasing human root dentin microhardness when compared with the group before treatment. Therefore, the authors rejected the null hypothesis that none of the materials studied would change the root dentin microhardness.

Although there is no clinical evidence that correlates a reduction in dentin microhardness with root fractures, this effect could cause a reduction in mechanical strength and cause the propagation of dentin microcracks [20]. 

The materials that increase dentin microhardness may be beneficial particularly in cases in which the dentin has become weakened (when antibiotic pastes or calcium hydroxide were used in revascularization procedure that diminished the dentin microhardness and fracture strength) [18, 21]. 

In the present study, the microhardness test was used to evaluate the effect of bioactive materials on root dentin. One of the advantages of these tests is that it provides the opportunity to observe the endodontic material at the healthy dentin interface where the material comes into contact with the substrate, with reliable results [22]. Although microhardness determination provides no specific information about the mechanical properties and dentin structures, it does provide indirect evidence of mineral loss or gain of dentin hard tissue [23]. 

In the present study, MTA showed the highest percentage increase of microhardness, similar to that of ERR, with the increase in percentage values of around 94% and 62% respectively. In the literature, reports that demonstrate the influence of dentin microhardness after the use of root repair materials are rare. 

**Table 1:** Distribution of Knoop microhardness (KHN) values (mean (SD)) of root dentin after use of repair cements and bioactive glasses and comparison with the respective controls (paired Student's t test

**Groups**	**Control**	**Experimental**	***P*** **-values** *
**MTA**	26.75 ( 9.42^B^)	46.82 (11.22^A^)	0.000
**ERR**	29.98 (9.65^B^)	44.3 (13.53^A^)	0.002
**45S5**	34.39 (12.67^B^)	43.66 (12.81^A^)	0.022
**NbG**	27.61 (10.2^B^)	37.06 (11.07^A^)	0.011

* *Different letters indicate statistically significant differences in the same row (P<0.05)*

**Table 2:** Comparison of the groups in relation to the percentage (%) of increase of the radicular dentin microhardness (mean (SD))

**Groups**	**Increase of microhardness (%)**	***P*** **-values** *
**MTA**	94.83 (42.68^ A^)	0.008
**ERR**	62.34 (39.88^ AB^)	
**45S5**	46.56 (30.05^ B^)	
**NbG**	53.84 (31.29^ B^)	

* *ANOVA one-way and Tukey’s test. Different letters indicate statistically significant differences between groups (P<0.05)*

The reports are restricted to evaluating only the microhardness of the reparative material alone and show that MTA presented higher microhardness values when compared with the other materials [24]. The high microhardness values found in the groups in which MTA was used may have been caused by its intrinsic hardness that would serve as support during the action of the microhardness test; this same reasoning could perhaps also explain the higher values of ERR.

On the other hand, White *et al.* [25] stated that the fracture resistance of bovine root dentin was diminished after 5 weeks of exposure to MTA. This may have occurred due to the capacity of MTA for degrading the collagen matrix of dentin over a prolonged period [26]. However, the quantity of collagen lost is limited to the surface in contact with the material [27]. Measurement of 20 µm from the root canal lumen could have avoided this problem. Therefore, the measurement of microhardness at this distance may have captured the mineral gain caused by the release of ions responsible for the increase in hardness [19].

However, when the roots of immature teeth were evaluated, these roots were observed to be less susceptible to fracture when calcium silicate-based materials substituted calcium hydroxide after prolonged contact with root dentin [26, 27]. 

In the present study, the bioactive glass pastes showed a lower increase in hardness (around 46% for 45S5 and 54% for NbG) when compared with MTA, but they were statistically similar to ERR. 

A flexural strength evaluation of dentin specimens stored in suspensions of 45S5 glass and calcium hydroxide demonstrated a reduction in strength of around 20% for the 45S5 glass, and 35% for calcium hydroxide [12]. However, according to the authors, all surfaces of the dentin specimens were exposed to the medication, and these restrictions indicated that the present results could not be extrapolated to the clinical situation. 

Bioactive glass (45S5) was originally developed as a bone conductive material; it may also react with saliva inducing the dissolution of ions Ca^++^, PO_4_^-3^ and Si^+4^ on the glass surface and subsequent precipitation of a polycondensated silica-rich layer (Si-gel) that serves as a template for the formation of calcium phosphate (Ca/P), which subsequently crystallizes into HCa [28]. The experimental bioactive glass containing niobium also presented characteristics similar to those of 45S5, but with a layer of niobium instead of silica, without compromising its bioactivity [29].

Many studies show that NbG bioactive glass has a capacity of releasing high amount of ions Ca/P [19], increase of pH [16], control of bacterial activity [14] and bioactivity when incorporated in gutta-percha and adhesive systems [14, 30, 31].

The finding that MTA showed a significantly higher percentage increase in hardness together with ERR leads to potential benefits in re-establishing the microhardness of weakened teeth. Although the bioactive glasses showed a lower percentage of increase in microhardness when compared with MTA, they may also be interesting for clinical use, considering that they increased the microhardness by approximately 50%, and have some advantages, such as not staining the dental structures.

## Conclusion

Within the limitations of this study, the results indicated that MTA increased the microhardness of root dentin to a significantly greater extent than did the bioactive glasses 45S5 and NbG. 
